# The Response of UV/Blue Light and Ozone Sensing Using Ag-TiO_2_ Planar Nanocomposite Thin Film

**DOI:** 10.3390/s19235061

**Published:** 2019-11-20

**Authors:** Tzu-Hsuan Lo, Pen-Yuan Shih, Chiu-Hsien Wu

**Affiliations:** 1 Department of Physics, National Chung Hsing University, Taichung 402, Taiwan; a0970712400@gmail.com (T.-H.L.); a234851678@gmail.com (P.-Y.S.); 2 Institute of Nanoscience, National Chung Hsing University, Taichung 402, Taiwan

**Keywords:** light sensor, SPR, composite, ppb-level ozone

## Abstract

We successfully fabricated a planar nanocomposite film that uses a composite of silver nanoparticles and titanium dioxide film (Ag-TiO_2_) for ultraviolet (UV) and blue light detection and application in ozone gas sensor. Ultraviolet-visible spectra revealed that silver nanoparticles have a strong surface plasmon resonance (SPR) effect. A strong redshift of the plasmonic peak when the silver nanoparticles covered the TiO_2_ thin film was observed. The value of conductivity change for the Ag-TiO_2_ composite is 4–8 times greater than that of TiO_2_ film under UV and blue light irradiation. The Ag-TiO_2_ nanocomposite film successfully sensed 100 ppb ozone. The gas response of the composite film increased by roughly six and four times under UV and blue light irradiation, respectively. We demonstrated that a Ag-TiO_2_ composite gas sensor can be used with visible light (blue). The planar composite significantly enhances photo catalysis. The composite films have practical application potential for wearable devices.

## 1. Introduction

Due to growing environmental awareness, photocatalysts have been identified as green materials for reducing air pollution [1]. Titanium dioxide (TiO_2_) is a popular photocatalytic material with considerable development potential [2,3]. With a bandgap of about 3.2 eV at an absorption wavelength of around 360 nm, TiO_2_ is a widely-studied n-type metal-oxide semiconductor (MOS) that is commonly used in photocatalysts and gas sensors [4,5,6]. Enhancing light response is an important application of the light-harvesting and gas sensing capabilities of TiO_2_. According to researchers, doping noble-metal particles can enhance the photocatalytic and degradation efficiency of TiO_2_ for chemical or biological matter. Absorption spectra also show good response in visible light when TiO_2_ nanocomposites (metal-doped TiO_2_) are used [7,8,9,10,11,12]. Several methods, such as metal particle doping, polymer nanocomposites, and core-shell nanoparticles [13,14,15,16], have been developed to enhance photocatalysis. The primary reason for this enhancement is that the surface plasmon resonance (SPR) produced by these metal nanoparticles can significantly change the visible light response and electrical properties of semiconductors. Via the SPR effect, the metal-nanoparticle composite provides additional electrons to the semiconductor. Different metal nanoparticles (Ag, Au) doped on MOSs are widely used in many fields to generate SPR and enhance photocatalysis; they are also used in gas sensors, environmental protection technologies, solar cells, energy storage devices, and photoelectric materials [17,18,19]. For example, researchers have applied SPR with magnetic microspheres for prion protein detection [20,21,22], a magnetic biochip (Au/Fe_2_O_3_/Au) for antigen detection [23], core-shell *γ*-Fe2O3@Au nanoparticles for low-field nuclear magnetic resonance [24], a gold film-coated side-polished fiber for temperature sensor fabrication [25], and Au@SiO_2_ core-shell NPs into TiO_2_ scaffold layer to increase the power conversion efficiency of solar cells [26].

With their excellent characteristics, MOSs doped with metal nanoparticles are highly desirable composite materials. In this study, we discuss the Ag-TiO_2_ planar composite film [17]. The methods used in the complexation of metal nanoparticles in TiO_2_ are primarily chemical-based. However, it is difficult to ensure the uniform doping of silver nanoparticles in TiO_2_ for large-scale and mass production [18,19,27,28,29]. We coated silver nanoparticles with TiO_2_ by electron-beam (e-beam) evaporation during the semiconductor fabrication process to ensure that the silver nanoparticles were in complete contact with the TiO_2_, without the use of high-temperature annealing, while still successfully sensing 100 ppb ozone [17]. This approach can significantly improve the practical application of silver particles in areas such as the manufacturing of gas sensors and wearable devices. We discuss the electrical properties, light response, conductivity change, and gas sensing of the Ag-TiO_2_ composite film for the detection of light and gas molecules.

## 2. Materials and Methods

We prepared the silver nanoparticles on a non-conductive glass and performed radio-frequency (RF)-magnetron sputtering at room temperature to deposit a silver film 10 nm thick. This film was then annealed at 250 °C for 1 h to produce nanoparticles. TiO_2_ film was overlaid with silver nanoparticles. Using a Ti_3_O_5_ tablet as a starting material, we then performed e-beam evaporation at a working pressure of approximately 0.1 Torr to produce TiO_2_ films with thicknesses of 10 nm, 20 nm, 30 nm, and 40 nm. The reaction equation is as follows [30,31]:

(1) $$2Ti_{3}O_{5}+O_{2}\to 6TiO_{2}$$

We labeled the Ag-TiO_2_ films with thicknesses of 10 nm and 20 nm as AT10 and AT20, respectively. We labeled the TiO_2_ films with thicknesses of 10 nm and 20 nm as T10 and T20, respectively. Using RF-magnetron sputtering, we deposited a gold film with a thickness of 100 nm on the sample as a measuring electrode. We used a multimeter (Keithley 2400) to measure the electrical properties. We determined the particle sizes, morphologies, and lattice structures of the silver nanoparticles by scanning electron microscopy (SEM) and X-ray spectroscopy, and measured the absorption spectra with a UV-Vis spectrometer.

In the experiments conducted to determine the light response and ozone detection, we used ultraviolet (UV) light and blue light-emitting diode as light sources. These measurements are described in detail elsewhere [32,33,34].

## 3. Results

### 3.1. Chacteristics of Ag-TiO_2_

**Figure 1:**
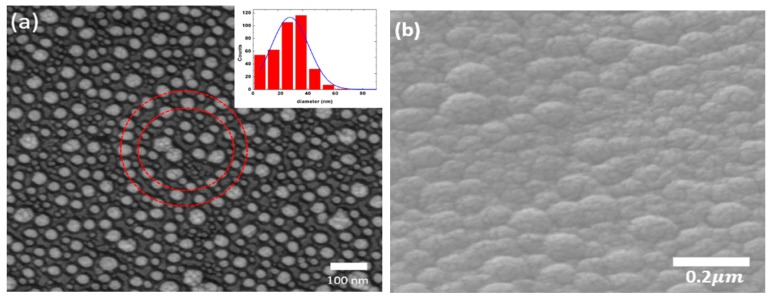
SEM images of the (**a**) Ag nanoparticles with the average size of 28 nm. The inset shows the distribution of particle sizes. (**b**) the Ag nanoparticles covering the TiO_2_ film film.

Figure 1 shows SEM images of the Ag nanoparticles and Ag-TiO_2_ film [35]. In Figure 1a, we can see that the Ag nanoparticles are almost spherical in shape. The silver nanoparticles have a uniform distribution with ring-like aggregates. There are four main nanoparticle groups with diameters of 5 nm, 15 nm, 25 nm, and 35 nm, respectively, and a maximum size of about 70 nm, as shown in the inset of Figure 1a. The average size is about 28 ± 13.26 nm. There are few particles whose sizes are over 80 nm. Figure 1b shows the Ag nanoparticles covering the TiO_2_ film; grains can be clearly observed on this Ag-TiO_2_ composition film, with average sizes ranging from 50 nm to 80 nm. This indicates that the particle sizes increased after the deposition of the 20 nm thick TiO_2_ film on the Ag nanoparticles. The image of the Ag-TiO_2_ composite film also shows an uneven surface.

**Figure 2:**
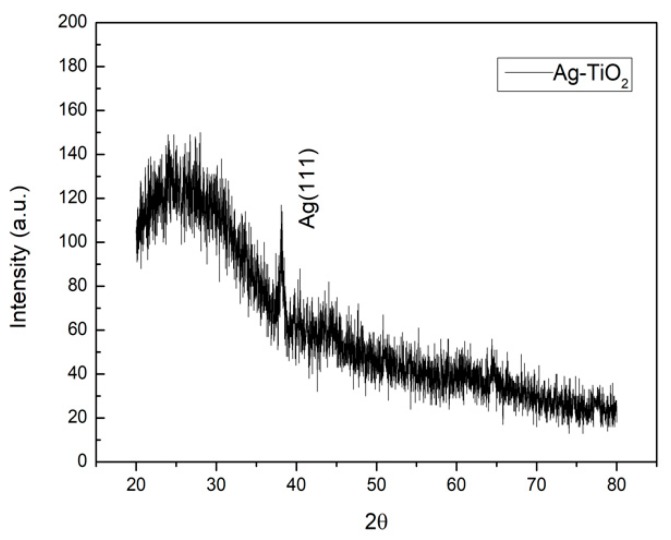
The X-ray diffraction pattern of the Ag-TiO_2_ composite.

Figure 2 shows the X-ray diffraction pattern of the Ag-TiO_2_ composite, in which we can see no distinct peak for the TiO_2_ film. The small peak at 2θ = 38.08 can be indexed as (111) for the silver nanoparticles. TiO_2_ films, without having undergone an annealing process by e-beam evaporation, are amorphous [3].

**Figure 3:**
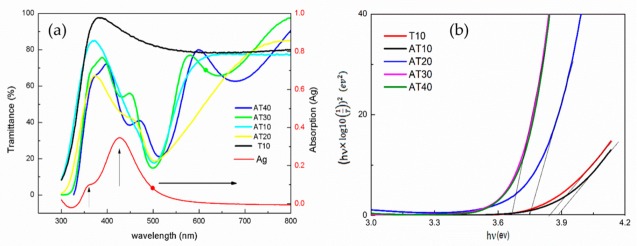
(**a**) The transmittance and absorption spectra of the samples. (**b**) The optical energy gap calculated by the Tauc equation.

Figure 3a shows the transmittance and absorption spectra of the samples (Ag, T10, AT10, AT20, AT30, and AT40), as measured by a UV-Vis spectrometer. The absorption spectrum of the silver nanoparticles alone (without TiO_2_ film) exhibits a clear and sharp peak at 429 nm, indicating silver spherical nanoparticles with the average sizes ranging from 30 to 50 nm [36,37]. This absorption spectrum indicates a localized SPR (LSPR) [38]. We can also see an unapparent peak at 360 nm in the absorption curve. The two resonance peaks in the UV-Vis spectra of the silver nanoparticles are mainly attributable to the dipole and multipole models (quadrupole) [39,40,41]. Multipole resonance is produced by a nonuniform electric field in the short-wavelength region, and it has been identified as hybrid quadrupole resonance. The main reason for the nonuniform polarization and electric fields is the nonlocal homogenized medium of the overlaid TiO_2_ film or the nanoparticle distribution [42].

The optical transmission spectra of T10 show that all of the TiO_2_ films were highly transparent, i.e., more than 80%, in the visible region. Both the pure T10 and AT10 nanocomposites exhibit transmission edges at about 350 nm. The transmission spectra of AT10 and AT20 reveal a redshift due to the influence of the added silver nanoparticles. The spectra of AT10, AT20, AT30, and AT40 exhibit transmission edges at ~330 nm and a broad wave around 500 nm [17,19,20,29,30]. The transmission spectra of the semiconductor and metal nanoparticles overlap, which indicates that the TiO_2_ and Ag metal nanoparticles were simultaneously excited by the light. There was also an edge at around 330 nm of the TiO_2_ film and a peak at 500 nm due to the SPR of isolated silver nanoparticles in the samples. We found the resonance peak of the silver nanoparticles to be significantly affected by the TiO_2_ film covering. The plasmon resonance of the silver nanoparticles was redshifted from a wavelength of 428 nm to 500 nm with TiO_2_ films of 10–30 nm. As the thickness of the TiO_2_ film increased to 40 nm, the redshift to 540 nm became more evident. The shift in the plasmon on this nanocomposite may be primarily attributable to the change in the permittivity of the medium. After coating, the oxide film has a much higher permittivity (*ε_m_*). This variety of plasmonic peak can be briefly explained by the Drude model [43]:

(2) $$W_{LSPR}\approx \frac{W_{P}}{\sqrt{1+2\varepsilon_{m}}}$$

where *W_LSPR_* is the frequency of the LSPR, *W_p_* is the plasma frequency of the bulky metal, and *ε_m_* is the dielectric constant of the medium. However, the shift values of the experimental data and those calculated by the Drude model are slightly different. This difference may be due to the size, shape, and distribution of the silver nanoparticles.

The UV-Vis transmission spectra of the Ag-TiO_2_ composites also reveal the transmittance to be less than 20%, which indicates that the composite films have a significant absorbance at the ~500-nm wavelength (blue light). The transmittance at wavelengths ranging from 300 to 600 nm is also less than 80%. This can be attributed to the fact that silver nanoparticles scatter the unabsorbed photons under light irradiation, resulting in an increase in the average photon path length, which increases the absorption [44]

Both the absorption peak of TiO_2_ and the Ag resonance showed a redshift, which indicates a reduction in the optical bandgap energy. The optical energy gap (*E_g_*) can be calculated using the Tauc equation:

(3) $${\left(\alpha hv\right)}^{\frac{1}{p}}=A\left(hv-E_{g}\right)$$

where *A*, *E_g_*, *h*, and *v* are constant, energy gap, plank constant, and frequency, respectively; *p* is the characteristic value of the optical absorption process, which is equal to 2 because TiO_2_ is an indirect energy gap material; and α is a coefficient. When the thickness of TiO_2_ was increased from 10 nm to 40 nm, the bandgaps of T10, AT10, AT20, AT30, and AT40 were 3.84 3.88, 3.75, 3.66, and 3.66 eV, respectively. The band gaps shown in Figure 3b are smaller than that of the 10 nm TiO_2_ film with increases in the thickness of the TiO_2_. The bandgap reduced no further when the TiO_2_ thickness was greater than the size of the Ag nanoparticles [45].

### 3.2. UV and Blue Light Response

**Figure 4:**
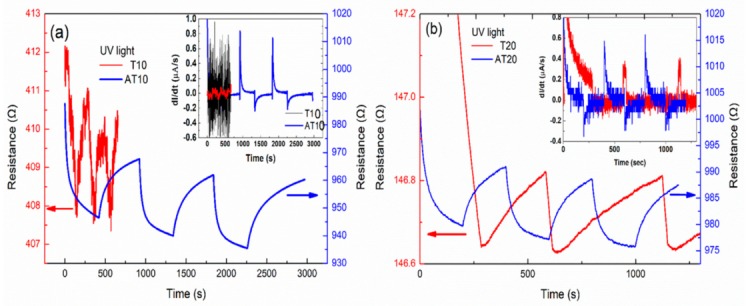
(**a**,**b**) show the resistance–time relation of Ag-TiO_2_ and TiO_2_ with different thickness under UV irradiation.

Figure 4a,b show the resistance–time relationship (bias: 1 V) of the Ag-TiO_2_ nanocomposite film (10 and 20 nm) and the TiO_2_ film (10 and 20 nm) under UV irradiation. Electrons and holes are generated when the films are irradiated by UV light, and the increased number of free electrons reduces the resistance. The light response of the composite was more significant than that of the TiO_2_ film, which means that the composite produced more electrons after UV irradiation [46].

An increase in the electrical conductivity of TiO_2_ under light irradiation and additional free carriers in the material can be generated in TiO_2_. The relationship of the resistance of the samples with and without light irradiation is as follows:

(4) $$R_{L}=R_{d}\left(\frac{\sigma_{d}}{\sigma_{d}+\Delta \sigma }\right),\;\frac{R_{d}}{R_{L}}=1+\frac{\Delta \sigma }{\sigma_{d}},$$

where *R_L_*, *R_d_*, *σ_d_*, and Δ*σ* are the resistance with light irradiation, resistance in the dark, the conductivity, and the conductivity change, respectively. According to the above equation, the percentages of Δ*σ*/Δ*σ_d_* for T10, T20, AT10, and AT20 for 50 s of light irradiation are 0.4%, 0.13%, 1.9%, and 1.1%, respectively. The value of Δ*σ*/Δ*σ_d_* for Ag-TiO_2_ is 4–8 times greater than that of TiO_2_, which means that the conductivity of the semiconductors was obviously improved by doping them with metal nanoparticles. The conductivity increase indicates that either the carrier density or carrier mobility increased.

The insets of Figure 4a,b show the relationship between the rate of photocurrent change and the detection time. The current density is given by J = σE. The conductivity is σ_0_ = n_0_eμ_0_, where n_0_ and μ_0_ are the carrier density and mobility in the dark, respectively. The conductivity (σ_L_) in light will increase to σ_L_ = σ_0_ + Δσ. The current change rate is given by dI/dt ∝ dσ/dt ∝ Δσ/Δt and is proportional to the carrier conductivity rate, where Δσ is attributed to the change in carrier density (Δn) and carrier mobility (Δμ). The resistance curves of AT10 and AT20 are more stable and significant than those of T10 and T20 (bias: 1 volt). The maximum current change rates of AT10 and AT20 are 0.7–0.8 and 0.4–0.5 (A/s), respectively, and are more significant than those of T10 and T20. The change is small for TiO_2_ under the same UV intensity. The differential curve fluctuated widely and was smoothed and averaged by several points. This variation is due to the SPR of the silver nanoparticles, whereby the silver nanoparticles provide TiO_2_ with hot electrons and reduce its optical band gap, thus generating more electrons in the conduction band of TiO_2_ with UV light irradiation.

The light response ((I_L_ − I_d_)/I_d_) of light for 50 s irradiation is shown in Table 1. Therefore, the photo response of the Ag-TiO_2_ composite is better than that of the TiO_2_ film. This reveals that the generation and recombination rates of electrons and holes in the Ag-TiO_2_ composite film are much greater and faster than those of the TiO_2_ film [46].

**Figure 5:**
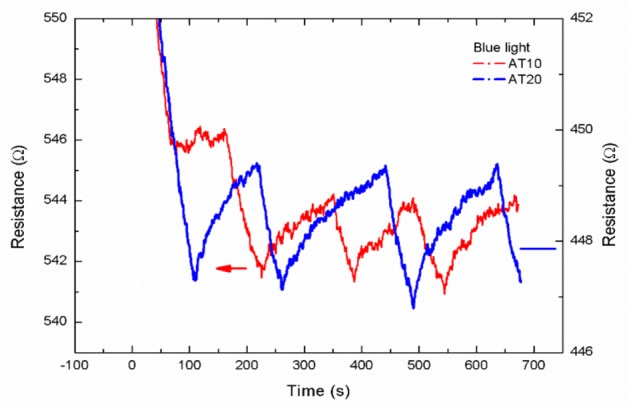
Ag-TiO_2_ composite film is irradiated by blue light.

When the Ag-TiO_2_ composite film is irradiated by blue light, the silver nanoparticles absorb the blue light to produce hot electrons for TiO_2_ and reduce the resistance value (Figure 5). This means that the composite film can absorb blue light. However, the 10 nm and 20 nm-thick TiO_2_ films showed no response to blue light.

### 3.3. Gas Sensing

**Figure 6:**
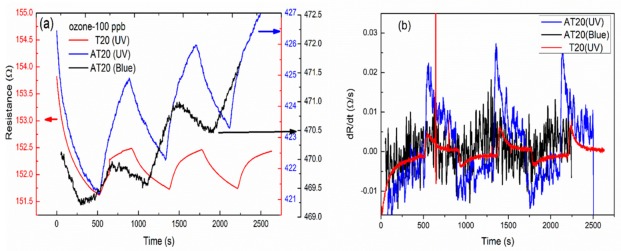
(**a**) The resistance–time relation of films at an ozone concentration of 100 ppb under different light irradiation. (**b**) Differential curves under light irradiation.

Ozone is known to be an oxidizing gas. Figure 6 shows the resistance–time relationship of films at an ozone concentration of 100 ppb, which is the index value indicating damage to human health. TiO_2_ is an n-type semiconductor, and we generated more electrons and holes by light irradiation [47,48]. The ozone concentration of the test box is simultaneously measured by a commercial ozone monitor (2B Tech 106-L) [32,33]. When strongly oxidizing O_3_ was introduced (25 °C and relative humidity ~45 ± 3%), adsorption and desorption reactions occurred simultaneously. Since an oxidizing gas was adsorbed on the TiO_2_, the free electrons were trapped, causing a decrease in the number of free electrons and an increase in resistance. In our experiment, UV and blue light were used to excite electrons, and thereby, facilitate gas absorption by the films. In Figure 6a, we can see a noticeable change in the resistance upon the introduction of ozone to the test chamber. There was little difference in the resistance changes of films under different types of light irradiation. Figure 6b shows the dR/dt versus time relationship of films under different types of light. The resistance changes of T20 (UV), AT20 (UV), and AT20 (blue) were 0.005, 0.03, and 0.02, respectively, at 100 ppb of ozone. Thus, the composite film AT20 exhibited a better response than the TiO_2_ film. The sensitivities of ozone for 300 s exposure are shown in Table 2. The sensitivity, i.e., AT20 (UV) > AT20 (Blue) > T20 (UV) >> AT20, revealed the good performance of the composite film with respect to ozone. The response of the composite film increased by roughly six and four times under UV and blue light irradiation, respectively. The silver nanoparticles enhanced the effectiveness of the transfer of free electrons from the conduction band of TiO_2_ to ozone. Thus, we demonstrated that the Ag-TiO_2_ composite gas sensor can be used with visible light. In Table 3, we summarize the sensitivities for ozone using different materials, as obtained by various research groups.

## 4. Conclusions

In this study, we successfully used a composite of silver nanoparticles and titanium dioxide film (Ag-TiO_2_). Ultraviolet-visible spectra revealed that silver nanoparticles have a strong SPR effect. In addition, we observed a strong redshift of the plasmonic peak when the silver nanoparticles covered the TiO_2_ thin film.

When measuring the light and gas responses, we found the light response of the composite film to be more versatile and responsive due to the SPR effect with UV irradiation. Under UV and blue light irradiation, the silver-nanoparticle electrons become excited and supplement those of the TiO_2_ film. The value of conductivity change for Ag-TiO_2_ is 4–8 times greater than that of TiO_2_ under light irradiation. We determined that the SPR effect provides additional electrons to the TiO_2_, thereby improving the light response and sensitivity of the gas sensor. We also found the conductivity of TiO_2_ to increase with Ag doping. In our experiments, the Ag-TiO_2_ nanocomposite film successfully sensed 100 ppb ozone. The gas sensor can also be operated with blue light; the results showed that the composite film could absorb blue light, and thus, can be used for application in different sensors such as gas sensors, light sensors, biosensors, and smart windows [53].

## Figures and Tables

**Table 1:** The response ((I_L_ − I_d_)/I_d_) of light for 50 s light irradiation.

	T10	AT10	T20	AT20
UV	0.2%	1.7%	0.1%	1.2%
Blue	X	0.45%	X	0.46%

**Table 2:** The sensitivity ((R_g_ − R_a_)/R_a_) of gas ozone for 300 s gas exposure.

	T20		AT20	
	Blue	UV	Blue	UV
Ozone	X	0.35%	0.38%	0.8%

**Table 3:** The response of ozone using different materials (R. T: Room temperature, * S = R_g_/R_air_, ^#^ S = (R_g_ − Rair)/Rair)**.**

Materials	Ozone (ppb)	Operating Temperature	Response	Reference
core–shell Au@TiO_2_	500	R. T	1.15 *	[16]
V_2_O_5_/TiO_2_	1000	300 °C	1.4 ^#^	[49]
Zn_0.95_Co_0.05_O	20	250 °C	0.4 ^#^	[50]
CuWO_4_	15	250 °C	~2 *	[51]
Pt/TiO_2_-SnO_2_	2500	R.T.(UV)	1100 *	[52]
Ag/TiO_2_	100	R.T. (Blue)	1.004 *	Present study
